# Book on the Physician Himself, and Things That Concern His Reputation and Success

**Published:** 1893-06

**Authors:** 


*Book on the Physician Himself, and Things that Concern his Reputation and Success.* By D. W. CATHELL, M.D. Tenth Edition. Pp. 343. Philadelphia: THE F. A. DAVIS CO. 1892.

The fact that this book has already run through ten editions proves that it has been favourably received by the profession. We are not surprised, therefore, to find that the whole volume is based on manly common-sense. Dr. Cathell begins with good advice to young practitioners as to the position and nature of their "offices," their dress and personal appearance, their medical education and general culture. He discusses questions of medical antagonism in a country where there is one doctor to 600 persons, instead of one to 1,650 in Great Britain. He urges attention to the code of ethics of the American Medical Association, and sets a high standard of purity, sobriety, patience, punctuality, and all the other virtues that justly inspire confidence in the profession of both countries. But besides inculcating these high principles, he does not neglect details on the advantages of youth and age in a doctor, on organisation, consultations, the demands of patients for false statements or wrong methods, on being chary with the speculum, on polypharmacy, over-specialism, professional reticence, and the minor duties of the doctor to the patient and the patient to the doctor. So much space is devoted to irregular practitioners that it is evident that they are more in the way of medical men in the States than in England.

Dividing gratuitous patients into God's poor, the devil's poor, and poor devils, he advises abstinence from the second class as private patients. And there are useful suggestions as to fees and bills, and means of self-preservation from patients who take undue advantage of their medical attendant.

Altogether, the book contains plenty of really valuable advice to every age and every grade of the profession; and it may be considered a typical handbook of all the honourable methods of medical life.

